# Environmental Life Cycle Assessment of Thermal Insulation Tiles for Flat Roofs

**DOI:** 10.3390/ma12162595

**Published:** 2019-08-15

**Authors:** Raul Gomes, José D. Silvestre, Jorge de Brito

**Affiliations:** CERIS, Instituto Superior Técnico, Universidade de Lisboa, Av. Rovisco Pais 1, 1049-001 Lisboa, Portugal

**Keywords:** C2C, “Cradle to gate”, environmental impacts, flat roofs, life cycle assessment, thermal insulation tiles, thermal insulation and protection products

## Abstract

Envelope insulation and protection is an important technical solution to reduce energy consumption, exterior damage, and environmental impacts in buildings. Thermal insulation tiles are used simultaneously as thermal insulation of the building envelope and protection material of under layers in flat roofs systems. The purpose of this research is to assess the environmental impacts of the life cycle of thermal insulation tiles for flat roofs. This research presents the up-to-date “cradle to gate” environmental performance of thermal insulation tiles for the environmental categories and life-cycle stages defined in European standards on environmental evaluation of building. The results presented in this research were based on site-specific data from a Portuguese factory and resulted from a consistent methodology that is here fully described, including the raw materials extraction and production, and the modelling of energy and transport processes at the production stage of thermal insulation tiles. These results reflect the weight of the raw-materials within the production process of thermal insulation tiles in all environmental categories and show that some life cycle stages, such as transportation of raw materials (A2) and packaging and packaging waste (A3.1 and A3.3, respectively), may not be discarded in a cradle to gate study of a construction material because they can make a significant contribution to some environmental categories. Moreover, complementary results regarding the economic, environmental, and energy performance Life Cycle Assessment (LCA) of flat roofs solutions incorporating the thermal insulation tiles studied showed that the influence of the economic costs on the total aggregated costs of these solutions is much higher than that of the environmental costs due to the lower environmental costs of the thermal insulation tiles at the product stage (A1–A3). These costs influenced the corresponding percentage of the environmental costs (between 14% and 18%) and the percentage of the economic costs (between 70% and 75%) in the total aggregated (environmental, economic, and energy) net present value (NPV). Finally, a complementary “cradle to cradle” environmental LCA discussion is presented including the following additional life cycle stages: maintenance and replacement (B2–B4), operational energy use (B6), and end-of-life stage and benefits and loads beyond the system boundary (C1–C4 and D).

## 1. Introduction

The consumption of energy worldwide contributes to increase pollution, environmental degradation, and greenhouse gases emissions. Globally, the building sector is responsible for a relatively large percentage (30% to 40%) of the overall primary energy consumption, and buildings are responsible for over 30% of the global CO_2_ emissions [1]. These figures are similar within the European Union (EU—see Table A1 in Appendix A for the list of abbreviations used in this paper), where buildings contribute to more than 40% of the overall energy consumption, also contributing in a considerable way (about 35%) to CO_2_ emissions [2]. Roofs, and particularly flat roofs, are one of the envelope’s building components that have an important role in terms of thermal exchanges with the exterior and therefore directly related with the energy consumption of the building. Moreover, flat roofs also influence the water-tightness of the building’s envelope. Considering the different layers of a flat roof system, thermal insulation tiles are used to insulate, preventing loses of heat by the roof, as finishing layer of the flat roof, and, simultaneously, to protect the under layers against mechanical and weather actions [3,4]. The use of this material results in a significant contribution to reduce the energy needed to preserve an adequate interior temperature and thus to accomplish energy efficiency [5] and reduce the environmental, economic, and energy impact of buildings [6]. A Life Cycle Assessment (LCA) investigation study was carried out to provide the environmental LCA of thermal insulation tiles for flat roofs produced in Portugal [7], and the corresponding results are presented in this research.

### 1.1. Thermal Insulation Tiles Studied

**Figure 1:**
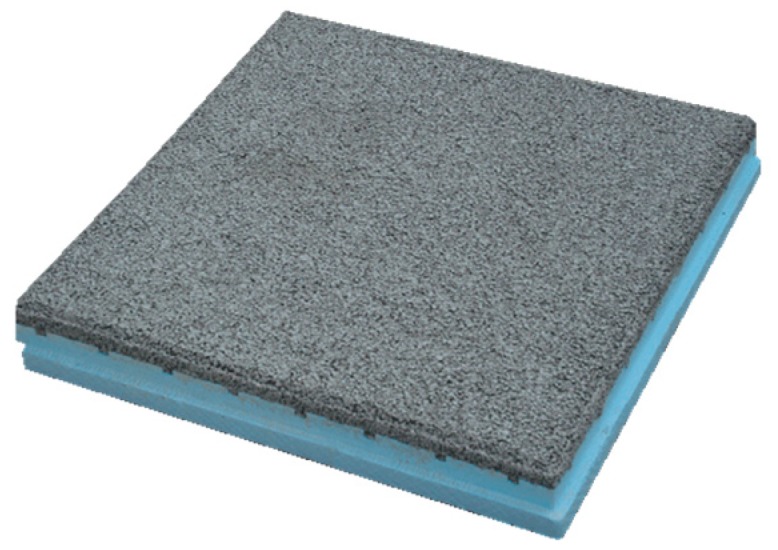
Thermal insulation tiles for flat roofs with an under layer of thermal insulation (extruded polystyrene—XPS) and a cement mortar layer on the top [8].

**Figure 2:**
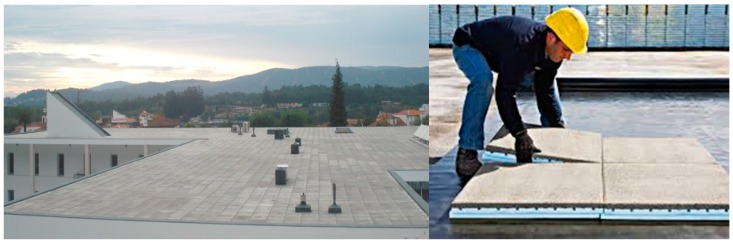
Inverted flat roof system (accessible to people) with heavy protection built with thermal insulation tiles for flat roofs (**on the left**). Laying prefabricated thermal insulation tiles over the waterproofing membranes (**on the right**) [8].

Thermal insulation tiles are, as stated, an insulation and (simultaneously) protection material that can be used in flat roofs of buildings. There are different types of these thermal insulation tiles available in the market with different brands, specifications, and purposes. The tile studied in this research is suitable to be used as a thermal insulation material and, simultaneously, as a heavy protection layer in flat roofs built by the inverted system (that is, using the insulation layer over the waterproofing membrane) (Figure 1 and Figure 2), since it integrates the thermal insulation layer of the flat roof system that, in this case, is placed over the waterproofing material.

The main technical characteristics of the thermal insulation tiles for flat roofs studied (dimensions, different thicknesses, density, thermal behaviour, and CE marking (standard)) are presented in Table 1. Different thicknesses of the insulation and mortar and different types of finishing allow for the production of different types of thermal insulation tiles. However, it was decided to study only one type of thermal insulation tile (called the default thermal insulation tile) with the following measures: length 600 mm, width 600 mm, and thickness 70 mm (corresponding to 40 mm of thermal insulation and 30 mm of mortar). This type of thermal insulation tile corresponds to almost all the production of the factory where data were collected during the period of the study.

Although the thermal insulation tiles studied in this research are produced in Portugal, there are other European countries where similar ones are produced. However, the authors were not able to identify, through an extensive literature review of research studies and Environmental Product Declarations (EPD) databases, any other LCA study for equal or similar products (not even in generic LCA databases), apart from the data presented in this research. Therefore, the LCA results for thermal insulation tiles for flat roofs released in this article, produced in a Portuguese factory (site-specific data from a national LCA study), are original at an international level. Moreover, because they were quantified using scientifically sound methods, in order to be clearly accepted at a worldwide level, they contribute to justify its innovation, and therefore this research.

This research is divided in this introduction plus four more sections. The second section describes the LCA methodology used. The third presents and analyses the resulting figures for the production of the thermal insulation materials and a complementary research study to assess the environmental, economic and energy LCA performance of flat roofs solutions integrating thermal insulation tiles in their composition. Section 4 presents a discussion of the methodology used, the results achieved, and some additional considerations regarding some use and end-of-life stages of thermal insulation tiles for flat roofs. Finally, the research ends by presenting the main conclusions that sum up the foremost findings of the work.

### 1.2. Goals and Scope

International standards on LCA [9,10] demands the need to clearly define the scope and the field of application of an LCA study. Therefore, the aim of the current LCA study is to outline the environmental profile of thermal insulation tiles for flat roofs manufactured in Portugal using site-specific data and publishing the results throughout the scientific community, mainly for those involved in the development of insulation and protection building products for flat roofs.

## 2. Materials and Methods

The applied LCA methodology is in accordance with the European standards developed under CEN/TC 350 (15804:2012+A1:2013 and EN 15978:2011 [11,12]), international standards on LCA [9,10], and other organizational procedures detailed described in this section.

### 2.1. Declared Unit Considered

As detailed in Figure 1, thermal insulation tiles are composed of two different materials: an insulation layer (XPS) and a cement mortar layer. Both of these components can have different thicknesses and technical characteristics, as presented in Table 1. Therefore, it was decided to consider a declared unit in this LCA study defined as one finished and packed thermal insulation tile with a width and length of (600 × 600) mm, an average weight of 23.05 kg, and a total thickness of 70 mm, of which 40 mm is from the thermal insulation element and 30 mm is from the cement mortar, and with an averaged thermal transmittance of 0.06 (W/(m.K)). This declared unit was defined considering the default thermal insulation tile produced in the Portuguese factory since this factory produces other thermal insulation tiles with different characteristics, as mentioned in Table 1. In this sense, the environmental impacts of this research are presented relatively to this material, since it is the reference used by the manufacturer to measure the manufacturing flows provided in the Life Cycle Inventory (LCI) filled.

### 2.2. System Boundaries Defined

Table 2 and Table 3 systematize the life cycle stages of construction materials and products in accordance with European Standards [11,12].

**Figure 3:**
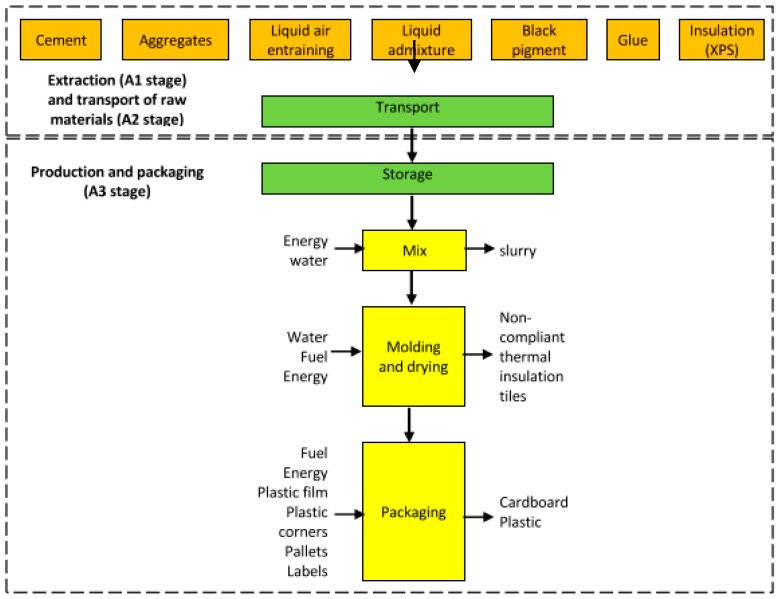
Key stages of thermal insulation tiles’ production and corresponding inputs and outputs.

Analysing Table 2, it is possible to conclude that the boundaries of a LCA system, for any LCA study of building materials or assemblies, can be organized in three different ways: from cradle to gate (including the product stage (A1–A3)); from cradle to grave (including in addition, the construction process stage (A4–A5), the use stage (B1–B7), and the end-of-life stage (C1–C4)); or from cradle to cradle (C2C) (which further includes the benefits and loads beyond the system boundary (D)) (Table 2 and Figure 3) [13,14]. The system boundaries are mandatory to define the unit processes to be included in the LCA study. In this research, it was used a cradle to gate LCA approach, therefore the environmental impact analysis starts at the A1 stage (extraction of raw materials) and continues throughout the A2 and A3 stages (transportation and storage of raw materials and production and packaging, respectively) (sub-stages highlighted in grey in Table 3). As detailed, the A3 stage was based on site-specific data from thermal insulation tiles for flat roofs collected from a Portuguese producer. In a complementary way, the authors present in Section 4.4, Section 4.5 and Section 4.6 further considerations about some other sub-stages of the thermal insulated tiles LCA (underlined in Table 3), namely sub-stages B2 and B4 (use stage—maintenance and replacement, in Section 4.4), B6 (use stage—operational energy use, in Section 4.5), and C1–C4 and D (end-of-life sub-stages and benefits and loads beyond the system boundary, in Section 4.6). These last stages of the LCA methodology can only be measured and evaluated based on scenarios [15,16].

### 2.3. “Cradle to Gate” Life Cycle Inventory (LCI) Considerations

Thermal insulation tiles for flat roofs are manufactured in an industrial plant situated about 150 km from Lisbon (the capital of Portugal). The study of this material began with some visits to the producer in order to allow the authors to understand the manufacturing process (A3) of the thermal insulation tiles studied and to prepare a questionnaire (LCI) to be filled with the main data related to it. The LCI questionnaire was interactively and iteratively prepared and sent to the manufacturer to register the production data necessary for the LCI modelling. The intermediate phases of the manufacturing process of thermal insulation tiles are identified in Figure 3. The LCI model is based on average production data from 2013. The background data for modelling the production process was based in data from Ecoinvent database (v3.2), available through the software tool used in this LCA study (e.g., data for the extraction/production of raw and packaging materials, electricity, and transportation of raw materials). As mentioned, all data used in the inventory phase was based on the answers that the producer provided in the questionnaire. The LCA tool chosen to model the production process was SimaPro (v8.4 of this software - PRé Sustainability, Amersfoort, Netherlands [17]).

The authors decided to subdivide the environmental impacts and potential benefits quantified in the A3 stage in order to make it easier to understand which was responsible for each environmental impact and taking into consideration the recommendations of European standards [11]. Thus, three independent information modules within the A3 stage were considered in this assessment, which set out the manufacturing process in more detail:

- A3.1—including manufacturing and transport to the factory of the packaging material that leaves the factory gate with the product;
- A3.2—including the gate to gate manufacturing of the product being studied and all internal transports;
- A3.3—including the production and disposal of raw materials or admixtures’ packaging and of the wrapping material of the packaging products.

The production of packaging for raw materials or admixtures (and also of the material for wrapping the packaging products) was included in the A3.3 module rather than the A3.2 (or A3.1) modules because the producer did not provide values in the LCI for each of the flows but, instead, provided them within the global packaging waste streams.

In order to assess the quality of the site-specific data achieved, a method developed by Ferrão was used [13]. This method (reproduced in a simplified way in Table 4) includes the most relevant indicators to evaluate the quality of data collected and the measure to be attributed to each parameter (1 to 5). The method was applied to measure the quality of the information used in this LCA study, and the conclusions are presented in the last row of Table 4 (with the corresponding measure indicated between brackets).

As mentioned before, the LCI data used in this study was collected from a Portuguese production plant, so it is therefore based on site-specific data. From Table 4, it is possible to conclude that the quality of the information of this study (with an average value of 2.0 in a 1 to 5 scale, where 1 = the best quality) can be considered good and adequate for the global purpose of this research work.

To sum up, the LCI was developed based on Portuguese site-specific data (for the manufacture site processes), and background processes were modelled based in modules from validated international databases (such as Ecoinvent and ELCD) during the modelling of the system process.

### 2.4. Selection of the Environmental Impact Assessment Method (EIAM) and Categories for the Study

European standard recommendations that provides the core product category rules for all construction products and services, EN 15804:2012 + A1 2013, defines the following seven impact categories to be chosen to assess the environmental impact of products, providing the EIAM CML-IA the corresponding characterization factors [7,11]. Therefore, because the SimaPro software chosen has CML-IA baseline (v4.3) available (developed in the Netherlands by the Institute of Environmental Sciences (CML) of the Leiden University), this EIAM was chosen for the impact assessment of the products studied, in the following seven categories:

- Global warming potential over a time span of 100 years—GWP;
- Ozone depletion—ODP;
- Acidification potential of soil and water—AP;
- Eutrophication potential—EP;
- Photochemical ozone creation potential—POCP;
- Depletion of abiotic resources (elements and fossil, separately, but the latter may be used and explained alone, if the values are known)—ADP (for elements) and ADP (f.f.) (for fossil).

In addition, the same standard also refers two additional environmental categories as mandatory for an LCA study, which allows us, in this case, to complementary study the environmental impacts directly on the consumption of primary energy (renewable and non-renewable) due to the production of the thermal insulation tiles studied. These two environmental categories were calculated based on a single indicator method expanded by PRé Consultants from the cumulative energy demand (CED) method [18]. They express the depletion of energy resources, and its calculation is based on the higher heating value [19]. In fact, the CED method provides results for six environmental categories (non-renewable, fossil; non-renewable, nuclear; non-renewable, biomass; renewable, biomass; renewable, wind, solar, geothermal; and renewable, water). In order to simplify the results, these categories were grouped and presented in the two mentioned categories with the same unit (megajoule—MJ). One expresses the consumption of primary energy, renewable (PE-Re), and the other the consumption of primary energy, non-renewable (PE-NRe).

## 3. Results and Interpretation

Thermal insulation tiles manufacturing process begins with the reception and storage of raw materials (Figure 3). All raw-materials except XPS and glue are mixed with water. At the same time, XPS is cut and prepared to be assembled with glue to the mortar mix during the moulding stage. After being regularised and pressed, the thermal insulation tiles are cured for three days and then packed and stored afterward. The LCA study of the above described manufacturing process allowed for the results presented below.

Therefore, this section presents the “cradle to gate” environmental impacts of thermal insulation tiles and some complementary results to assess the economic, environmental, and energy life cycle performance of different flat roofs solutions integrating the thermal insulation tiles studied as insulation and protection material.

Furthermore, Section 4 complements this section, with an additional discussion regarding the “cradle to cradle” environmental LCA of thermal insulation tiles for flat roofs, including their expected service life and environmental impacts at stages C1–C4 and D, as well as the influence in the expected operational energy use of considering the better thermal insulation of these tiles.

### 3.1. Cradle to Gate (A1–A3) Environmental Impacts Results

**Figure 4:**
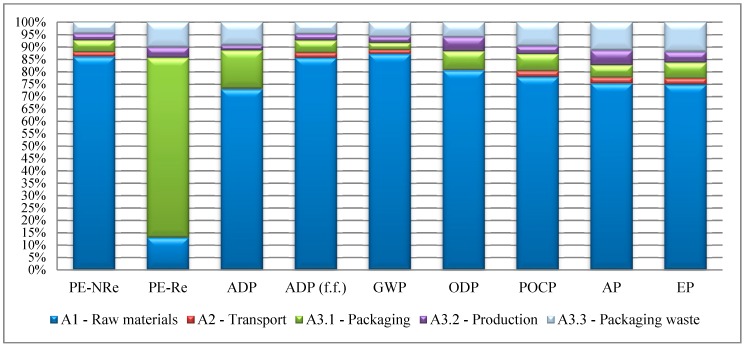
Contribution of each sub-stage of thermal insulation tiles production for environmental impacts.

Figure 4 shows the relative contribution (in percentage) of each sub-stage (A1–A3) to the cradle to gate environmental impacts of thermal insulation tiles for flat roofs and reflects the weight of the raw materials within the production process of these products in all environmental categories (A1 has a contribution of 73% or more to all environmental impact categories except PE-Re). Within the raw materials extraction and transportation to the factory (A1–A2), the insulation material (XPS) gives the highest contribution to the environmental impacts in the PE-Re and ADP (f.f.) categories (56.1% in PE-Re—biomass; 74.6% in ADP (f.f.)) and cement in GWP (60%) comparatively to the others, and cement is the second one with a contribution of 42.8% in PE-Re biomass and 20.2% in ADP (f.f.), and XPS with 36.7% in GWP. In terms of PE-Re, it is also possible to see the contribution of the packaging of the thermal insulation tile (A3.1) (73%) and of the disposal of raw materials or admixtures’ packaging, mainly due to the use of wood pallets (A3.3) (10.1%), and this impact is also significant in terms of AP (11.4%) and EP (12%). Figure 4 also shows that the A3.2 sub-stage only has a significant impact on ODP and AP (both 6%) mainly due to electricity consumption during the manufacturing process [7].

Cradle to gate LCA results of the production of one default thermal insulation tile are presented in Table 5.

### 3.2. Environmental, Economic, and Energy C2C Life Cycle Performance of Flat Roof Solutions with Thermal Insulation Tiles in their Composition

As mentioned, thermal insulation tiles are suitable to be used in inverted flat roofs systems accessible to people, where thermal insulation layers are over the waterproofing layer. Table 6 presents some of the main characteristics and compositions of inverted flat roof solutions, integrating thermal insulation tiles, considered in a complementary study performed that assessed the environmental, economic, and energy performance of 114 different solutions (also integrating other materials than thermal insulation tiles in the insulation and protection layers) for flat roofs. Although not being the main focus of this paper, the results showed that the environmental performance of flat roofs with thermal insulation tiles is generically better than those solutions using XPS and EPS as insulation layer and other paving products in the protection layer in almost all environmental impact categories, mainly due to lower environmental impacts at the product stage (A1–A3) of these solutions (for the same use of the flat roof solutions studied, namely, inverted solutions accessible to people) [7]. The opposite happened regarding the energy needs (B6), demonstrating that these type of solutions have higher values than the alternatives studied. However, solutions with thermal insulation tiles had a better economic performance when considering their initial acquisition cost (in year 0) (A1–A5) and their maintenance needs (C2–C4) [7].

Aggregating the three analysed dimensions (environmental, economic and energy) using the same economic unit (using the 3E *cost*-C2C methodology [20]), it was possible to conclude that the influence of the economic costs on the total aggregate costs is much higher than that of the environmental costs due to lower environmental costs of the thermal insulation tiles at the product stage (A1–A3). These costs influenced the corresponding percentage of the environmental costs (between 14% and 18%) and the percentage of the economic costs (between 70% and 75%) in the total aggregated (environmental, economic and energy) NPV (net present value) [7].

These results occurred in a very similar way to other alternative solutions within this type of flat roofs, not integrating thermal insulation tiles, where economic costs also had a high influence (63% to 71%) within the aggregated costs, mostly due to the acquisition market costs (in year 0) (with a influence of 64% to 75% on the total economic costs) [7].

## 4. Discussion

This section complements the previous one by presenting a discussion of the methodology and options considered and of the results achieved in this research, namely for the product stage (A1–A3) of the thermal insulation tiles. Moreover, an additional discussion is presented regarding some other stages of the C2C LCA of thermal insulation tiles, namely use and end-of-life stages.

### 4.1. A1–A3—Cradle to Gate (A1–A3) Environmental Impacts Discussion

The authors performed extensive research in international scientific literature databases searching for similar studies that could be used to compare and validate the results presented in this research. However, it was not possible to find any comprehensive LCA study accessible worldwide regarding thermal insulation tiles apart from the one presented in detail in this research. Therefore, the LCA results presented in this research are original and innovative both in national and international terms, since no LCA data set was up to now acknowledged for very similar (nor similar) products. In this case, the extraction of the raw materials for the production of the thermal insulation tiles—cement (two types), aggregates (two types), liquid air entraining, liquid admixture, black pigment, glue, and XPS—were modelled using different processes from Ecoinvent, from the European database ELCD (including all transportation processes) and data from two European Federation of Concrete Admixtures Associations (EFCA) EPD. The processes and data used were mainly referred to European region although one of the aggregates is referred to RoW (rest of the world).

Other research studies showed that raw material extraction and processing (or of secondary products input) (A1) have a relevant influence in the cradle to gate environmental impacts of several insulation materials [5] and cement-based products [7], which was confirmed in the LCA results of the thermal insulation tiles studied (Figure 4) in all environmental categories except in PE-Re (with only 13%). The LCA results of the thermal insulation tiles “product stage” also show that some life cycle stages, such as transportation of raw materials (A2) and packaging and packaging waste (A3.1 and A3.3, respectively), may not be discarded in a cradle to gate study of a construction material because they can make a significant contribution to some environmental categories (Figure 4).

### 4.2. A2—Transport to the Manufacturer Modelling

The transportation processes were modelled based on the producer information (i.e., type of transport used, average distances, type of trucks, and load factors, when applicable). When this data was not specified in the LCI, generic data was used, mainly from European databases. Raw materials or admixtures’ packaging and wrapping material of packaging products transportation was not considered due to the low impact of these processes on the total environmental impacts and the difficulty of isolating them from the total packaging waste generating during the manufacturing process of the thermal insulation tiles [7]. Data from the ELCD database were used to model the ground transportation processes because the majority of raw materials are produced in Europe (hence, the majority of the transportation procedures were carried out inside Europe) and because it has a more realistic approach to reality since it considers a load factor of 85% [7,21,22].

### 4.3. A3.2—Energy Processes Used

The Ecoinvent database [21] includes processes for different energy carriers, suitably representing the reality of Western countries, including Portugal. It also considers the co-dependent network between countries that symbolizes the international trade in electricity [23]. For that reason, these data were chosen as the basis to model the energy supply of the manufacturing processes studied, although the corresponding quantification was modelled using site-specific data. The Ecoinvent database includes processes that represent the national electricity supply for industrial (Electricity, medium voltage [PT], market for, alloc Rec S) Portuguese consumers, based on the energy mix of 2012, updated in 2016 [17,24]. This process represents the supply of 1 kWh of medium voltage electricity and includes data related with Portuguese energy production (or imported) after being transformed to medium voltage, supply network, air emissions, and energy losses during the supply processes. Although the manufacturing process (A3) of thermal insulation tiles is not energy-intensive, using the Portuguese electricity mix that expresses the current status is essential to accurately estimate the environmental impacts within the LCA methodology.

### 4.4. B2–B4—Maintenance and Replacement of Thermal Insulation Tile for Flat Roofs

The lowest durability (or predictable service life) of thermal insulation tiles was projected as 50 years without affecting its thermal characteristics, and therefore its thermal performance in the protection action that it confers to flat roofs under layers [7,25]. This expected life service was based on the expected service life of insulation (namely XPS) and cement-based materials, which are considered to be similar to the building service life (50 years). This was also the service life defined for the declared unit of the complementary study (presented in Section 3.2) that assessed the economic, environmental, and energy performance of flat roof alternatives solutions using thermal insulation tiles (and other materials) as protection layer [7,25].

Therefore, the expected service life of the waterproofing layer (placed under the thermal insulation tiles) affects the number of maintenance actions needed over the flat roof systems [25]. Moreover, the use of thermal insulation tiles in an inverted flat roof system extends the expected service life time of the waterproofing membrane, thus reducing the number of maintenance operations needed for the flat roof system [7].

However, because a 50-year service lifespan was established for the thermal insulation tiles, it was considered that maintenance actions of the waterproofing layers would not influence the maintenance of the thermal insulation tiles, since they are cautiously removed by hand to allow for the replacement of the waterproofing system and, afterward, carefully replaced by hand in order to make sure that they are not damaged by these operations.

Therefore, the authors decided not to consider any replacement of the thermal insulation tiles throughout the 50 years of service life of a building, nor their maintenance, in the research mentioned in Section 3.2.

### 4.5. B6—Use Stage—Operational Energy Use Considerations

Using thermal insulation tiles with different thicknesses of the insulation layer (XPS) can decrease the operational energy needed during the use stage of the building due to the low thermal conductivity of XPS, which is lower than that of other alternative products with the same thickness (for example, expanded polystyrene—EPS or insulation cork board—ICB) [26]. However, the quantification of this advantage of XPS would only be possible by performing a comparison of the environmental, economic, and energy performance of insulation materials used in the building’s envelope. This research can be done using a specific building and different insulation materials applied in an external assembly (either in the vertical or horizontal building envelope) and performing a thermal simulation of the performance of the building for a complete cycle (for example, one year) to calculate the corresponding heating and cooling needs (applying the Portuguese regulation) and then using the 3E-C2C method (mentioned in Section 3.2) to perform an assessment of the life cycle performance from cradle-to-cradle of these assemblies in these three dimensions [20,27]. This was the methodology applied in Section 3.2, as mentioned, to perform an environmental, economic and energy LCA assessment of the performance of 114 different flat roofs solutions integrating different materials (including XPS, low-density and high-density EPS boards, and MW as insulation materials) in their composition [7].

### 4.6. C1–C4—End-of-Life Stage and D—Benefits and Loads Beyond the System Boundary Modelling

The environmental impacts and loads of thermal insulation tiles at stages C1–C4 and D depend on the scenario defined for the end of life of this material. This stage is, probably, the most complex within the life cycle of flat roofs, considering the uncertainty associated to this life cycle stage that will happen in a distant future [28,29]. Moreover, this life cycle stage only represents less than 1% of the C2C environmental impacts of construction products [20]. Therefore, this section describes the procedure for the calculation of these impacts but not the actual values.

Demolition of flat roofs (or of their elements) happens when these systems are not complying with the functional requirements defined by national or international standards. The last stage of the life cycle of flat roofs can include demolition (selective or undifferentiated) and processing and recycling of resulting waste. If selective demolition is considered, it must include removing the thermal insulation tiles from the flat roof system and delivering them to a recycling site in order to allow processing the mortar and insulation wastes separately [7]. The environmental impacts that must be calculated, include:

- The demolition environmental impacts (sub-stage C1);
- The transportation to waste processing (C2);
- The waste processing for reuse, recovery and/or recycling (C3);
- The waste disposal (C4);
- The reuse, recovery and/or recycling potential (D).

The environmental impacts of the transport and disposal of the Construction Demolition Waste (CDW) can be based on Portuguese case studies that used data from waste and recycling operators [7,25]. In this research, transport data of waste provided by the producer was considered in the modelling of the referred disposal of each waste stream (modelled with waste treatment processes from Ecoinvent) generated during the product stage (A1–A3).

## 5. Conclusions

The cradle to gate LCA results per life cycle stage and environmental category of thermal insulation tiles are presented and analysed in this research, along with the identification of the processes that have a significant contribution to each environmental impact category. Moreover, a complementary environmental, economic, and energy LCA performance of different flat roofs solutions integrating the thermal insulation tiles studied is presented with the main purpose of assessing the contribution of this material to the performance of the studied solutions in the three dimensions mentioned.

In this study, the latest information available on the Portuguese electricity mix was considered in order to accurately estimate the environmental impacts of the company arising from the consumption of energy for the manufacture of these tiles.

The LCA of the production of thermal insulation tiles shows that the most important stage is A1—raw materials production, with a contribution of 73% or more to all environmental impact categories except PE-Re. Within the raw materials extraction and transportation to the factory (A1–A2), the insulation material (XPS) gives the highest contribution to the environmental impacts in the PE-Re and ADP (f.f.) categories and cement in GWP (60%), and cement is second, with a contribution of 42.8% in PE-Re—biomass and 20.2% in ADP (f.f.), and XPS with 36.7% in GWP. It was also found that some life cycle stages, such as transportation of raw materials (A2) and packaging and packaging waste (A3.1 and A3.3, respectively), may not be discarded in a cradle to gate study of a construction material because they can make a significant contribution to some environmental categories. Moreover, complementary results regarding the economic, environmental, and energy performance Life Cycle Assessment (LCA) of flat roofs solutions incorporating the thermal insulation tiles studied showed that the influence of the economic costs on the total aggregated costs of these solutions is much higher than that of the environmental costs due to the lower environmental costs of the thermal insulation tiles at the product stage (A1–A3). These costs influenced the corresponding percentage of the environmental costs (between 14% and 18%) and the per-centage of the economic costs (between 70% and 75%) in the total aggregated (environmental, economic, and energy) net present value (NPV).

The LCA results described in this research are scientifically comprehensive since they were reached throughout a reliable methodology (described in detail in this research) following European standards. These results are innovative and up-to-date for this specific construction material that aggregates technical characteristics that allow its simultaneous application as insulation and protection material in flat roof solutions of buildings.

Furthermore, these results include for the first time the “cradle to gate”, and some considerations regarding the “cradle to cradle”, environmental performance of thermal insulation tiles.

## Figures and Tables

**Table 1:** Most relevant technical characteristics of the thermal insulation tiles for flat roofs studied.

Layer	Size in Plan View (mm x mm)	Thickness [mm]	Finishing	Density [kg/m^3^]	Thermal Conductivity—λ [W/(m.K)]	CE Marking (Standard)
Mortar	600 × 600	20–40	P2—Porous, with aggregates from 2 to 4 mm in the mortar S—Simple, with aggregates from 1 to 2 mm in the mortar	P2—1820; S—1860	1.80	No
Insulation (XPS)		30–100		32	0.035	

**Table 2:** Life cycle stages of building materials classification based on European standards, including the corresponding system boundaries [11].

System Boundaries	Cradle to Cradle				
	Cradle to Grave				Benefits and Loads Beyond the System Boundary (D)
	Cradle to Gate	Gate to Grave			
LCA information modules		Product stage (A1–A3)	Construction process stage (A4–A5)	Use stage (B1–B7)	End-of-life stage (C1–C4)

**Table 3:** Detailed description of the life cycle stages of building materials modules (assessed in this research—highlighted in grey; only discussed—underlined) [11,12].

LCA Information Modules	Life Cycle Stage Designation and Description	
Product stage (A1–A3)	A1	Raw material extraction and processing, processing of secondary material input
	A2	Transport to the manufacturer
	A3	Manufacturing
Construction process stage (A4–A5)	A4	Transport to the building site
	A5	Installation in the building
Use stage (B1–B7)	B1	Use or application of the installed product
	B2	Maintenance
	B3	Repair
	B4	Replacement
	B5	Refurbishment
	B6	Operational energy use
	B7	Operational water use
End-of-life stage (C 1–4)	C1	De-construction, demolition
	C2	Transport to waste processing
	C3	Waste processing for reuse, recovery and/or recycling (3R)
	C4	Disposal
Benefits and loads beyond the system boundary (D)	D	Reuse, recovery and/or recycling (3R) potential

**Table 4:** Estimation of the quality of the information used in the Life Cycle Assessment (LCA) study and in the Life Cycle Inventory (LCI) of the thermal insulation tiles studied (adapted from [13,7]).

Measure to Be Attributed	Sureness	Reliability	Temporal correlation	Geographic Correlation	Technological Correlation
1	Confirmed data and based on measurements	Data from a sufficient number of companies during a considerable period of study	Data not superior than 3 years from the year under study	Data from the area under study	Data from the manufacture under study
2	Partially confirmed data and based on hypothesis or, not confirmed but based on measurements	Data from a small number of companies, but for adequate periods	Data less than 6 years	Average data from a larger area than that region studied, but including the studied region	Data from the same processes/materials but from other manufacturers
3	Not confirmed data and partially based on hypothesis	Data from a suitable number of companies, but for short periods	Maximum difference of 10 years	Data from an area with similar production conditions	Data from the same processes/materials but from a different technology
4	Confirmed or qualified estimations (produced by experts)	Representative data but from a small number of companies and from short periods, or incomplete data from an adequate number of companies and period durations	Data less than 15 years	Data from a geographical area with production conditions with some similarities	Data from similar processes/materials but analogous technology
5	Neither confirmed nor qualified data estimations	Unknown reliability, or incomplete data from a small number of companies and/or short periods	Unknown age of data or data above 15 years	Data from an unknown area	Data from similar processes/materials but different technology
**Quality of the information from the company that produces the thermal insulation tiles (Average 2.0)**	**Unverified (but including a visit to the production line) but based on measurements (2)**	**Representative data but from one company and from one year of production; market share (40%)—most important company in the national market (4)**	**Year of the LCI—2013 (2)**	**Data from the area under study (1)**	**Data from the manufacturer under study (1)**

**Table 5:** LCA results for each sub-stage of the “product stage” (A1–A3) of one default thermal insulation tile for flat roofs.

Category Indicator	Unit	Life Cycle Stages (Total per Default Thermal Insulation Tile)					
		A1–A3	A1	A2	A3.1	A3.2	A3.3
PE-NRe	MJ	6.98E + 01	6.01E + 01	1.29E + 00	3.38E + 00	1.86E + 00	3.20E + 00
PE-Re	MJ	1.32E + 01	1.72E + 00	1.72E − 03	9.56E + 00	5.55E − 01	1.33E + 00
ADP	kg Sb eq	2.78E − 06	2.03E − 06	3.63E − 09	4.32E − 07	5.64E − 08	2.59E − 07
ADP (f.f.)	MJ	6.38E + 01	5.46E + 01	1.28E + 00	3.15E + 00	1.67E + 00	3.05E + 00
GWP	kg CO_2_ eq	5.51E + 00	4.80E + 00	9.11E − 02	1.56E − 01	1.40E − 01	3.20E − 01
ODP	kg CFC-11 eq	1.85E − 07	1.49E − 07	1.85E − 10	1.41E − 08	1.06E − 08	1.11E − 08
POCP	kg C_2_H_4_ eq	1.24E − 03	9.70E − 04	2.91E − 05	8.60E − 05	3.92E − 05	1.20E − 04
AP	kg SO_2_ eq	1.65E − 02	1.24E − 02	4.09E − 04	8.08E − 04	9.86E − 04	1.88E − 03
EP	kg PO_4_^3−^ eq	3.81E − 03	2.85E − 03	9.35E − 05	2.44E − 04	1.67E − 04	4.56E − 04

**Table 6:** Example of different flat roofs solutions (with thermal insulation tiles) studied in a complementary research to assess the environmental, economic and energy performance of 114 flat roofs solutions (i.e., solutions for inverted flat roofs accessible to people) [7].

Shaping-Layer	Waterproofing Layer	Insulation Layer			Protection Layer	U-Value W/(m^2^.°C)	Total Thickness (mm)
		Material	λ (W/m.°C)	Thickness (mm)			
Lightweight concrete with EPS regranulate	APP bituminous membrane	XPS	0.036	80	Thermal insulation tiles	0.18	470
	SBS bituminous membrane						
	TPO thermoplastic membrane						
	PVC thermoplastic membrane						

## Appendix A

**Table A1:** The following abbreviations are used in this manuscript

Abbreviations	Full Name
AP	Acidification Potential
ADP	Abiotic Depletion Potential
C2C	cradle to cradle
CDW	Construction and Demolition Waste
CED	Cumulative Energy Demand
EIAM	Environmental Impact Assessment Method
EP	Eutrophication Potential
EPD	Environmental Product Declaration
EPS	Expanded Polystyrene
EU	European Union
GWP	Global Warming Potential
ICB	Insulation Cork Board
LCA	Life Cycle Assessment
LNEC	National Civil Engineering Laboratory
ODP	Ozone (stratospheric) Depletion Potential
PE-NRe	Consumption of primary energy, non-renewable
PE-Re	Consumption of primary energy, renewable
POCP	Photochemical Ozone Creation Potential
XPS	Extruded Polystyrene
